# Morphologically indistinguishable hybrid *Carassius* female with 156 chromosomes: A threat for the threatened crucian carp, *C*. *carassius*, L

**DOI:** 10.1371/journal.pone.0190924

**Published:** 2018-01-23

**Authors:** Martin Knytl, Lukáš Kalous, Kateřina Rylková, Lukáš Choleva, Juha Merilä, Petr Ráb

**Affiliations:** 1 Department of Cell Biology, Faculty of Science, Charles University, Prague, Czech Republic; 2 Department of Zoology and Fisheries, Faculty of Agrobiology, Food and Natural Resources, Czech University of Life Sciences Prague, Prague, Czech Republic; 3 Department of Genetics and Breeding, Faculty of Agrobiology, Food and Natural Resources, Czech University of Life Sciences Prague, Prague, Czech Republic; 4 The Czech Academy of Sciences, Institute of Animal Physiology and Genetics, Laboratory of Fish Genetics, Liběchov, Czech Republic; 5 Department of Biology and Ecology, Faculty of Science, University of Ostrava, Ostrava, Czech Republic; 6 Ecological Genetics Research Unit, Department of Biosciences, University of Helsinki, Finland; Southwest University, CHINA

## Abstract

The crucian carp *Carassius carassius* (Linnaeus, 1758), is native to many European freshwaters. Despite its wide distribution, the crucian carp is declining in both the number and sizes of populations across much of its range. Here we studied 30 individuals of a putative pure population from Helsinki, Finland. Despite clear external morphological features of *C*. *carassius*, an individual was of a higher ploidy level than the others. We therefore applied a set of molecular genetic (S7 nuclear and cytochrome *b* mitochondrial genes) and cytogenetic tools (sequential fluorescent 4’, 6-diamidino-2-phenylindole [DAPI], Chromomycin A_3_ [CMA_3_], C-banding and *in situ* hybridization [FISH] with both 5S and 28S ribosomal DNA probes) to determine its origin. While all examined characteristics of a diploid representative male (CCAHe2Fi) clearly corresponded to those of *C*. *carassius*, a triploid individual (CCAHe1Fi) was more complex. Phylogenetic analysis revealed that the nuclear genome of CCAHe1Fi contained three haploid sets: two *C*. *gibelio* and one *C*. *carassius*. However the mitochondrial DNA was that of *C*. *gibelio*, demonstrating its hybrid origin. The FISH revealed three strong (more intensive) 5S rDNA loci, confirming the triploid status, and an additional 24 weak (less intensive) signals were observed in the chromosome complement of CCAHe1Fi. On the other hand, only two strong and 16 weak 5S rDNA signals were visible on the chromosomes of the CCAHe2Fi male. 28S rDNA FISH revealed four strong signals in both CCAHe1Fi and CCAHe2Fi individuals. CMA_3_ staining revealed four to six CMA_3_-positive bands of CCAHe1Fi, while that of diploids contained only two to four. The fact that a polyploid hybrid *Carassius* female with a strong invasive potential may share morphological characters typical for endangered *C*. *carassius* highlights a need to combine genetic investigations of *Carassius* cryptic diversity with conservation measures of *C*. *carassius* in Europe.

## Introduction

Hybridization in the broad sense is considered as a transmission of alleles among the genomes of related species [1]. Hybridization has been recognized as a process of considerable importance for species’ evolution [2], as it can often lead to incomplete reproductive isolation. This is especially true for species like fishes that use external fertilization and experience spatial and/or temporal overlap in spawning habitats. Cyprinids in particular are known to experience hybridization between closely related species [3–5].

Natural heterogeneity in aquatic habitats promotes and maintains diversification, which is important for speciation and the course of evolution. However, human activities can lead to a reduction in such habitat variation. These anthropogenic alterations often provide favourable conditions that promote hybridization between native taxa [6,7]. In addition, the number of non-native fish species in freshwater ecosystems is increasing at a global scale e.g. [8], and introduction of non-native species has been recognised as one of the major causes of the worldwide decline in aquatic fauna diversity [9,10]. Similarly, hybridization between native and non-native taxa is recognised as a significant driver of biodiversity loss [11].

The genus *Carassius* (Nilsson, 1832), a member of an ancient paleotetraploid clade Cyprinini [12], includes at least four recognized species, as well as some forms with unclear taxonomic status. The crucian carp *C*. *carassius* (Linnaeus, 1758), is native to much of Europe. Despite its wide distribution, the number and sizes of crucian carp populations are declining across much of its range [13,14]. For example, the most recent Red List of threatened species in the Czech Republic now lists *C*. *carassius* in the Critically Endangered category (CR, A2ace; [15]). Because some local populations have experienced recent extinctions, e.g. [16,17], awareness of the threats to *C*. *carassius* is appropriately building [14]. Non-native invasive *Carassius* taxa expand in Europe and pose a growing threat to *C*. *carassius* [18]. The mitochondrial lineages of *C*. *gibelio* (Bloch, 1782), goldfish *C*. *auratus* (Linnaeus, 1758) and *C*. *langsdorfii* (Temminck & Schlegel, 1846) have been recognized within the *C*. *auratus* complex *sensu* Takada et al. [19] in European waters [17]. Their chromosome numbers are both 2n = 100, and 3n ≈ 150 [20–23], while *C*. *carassius* possesses 2n = 100 e.g. [24,25]. The “triploid” chromosome numbers (3n ≈ 150) range from 141 [26] to 166 [27].

A well-documented case of hybridization is that seen between *C*. *carassius* and feral *C*. *auratus*, which originated in the Far East [17,28]. Several other hybrids between *C*. *carassius* and introduced *C*. *gibelio* and *C*. *auratus* have been discovered using microsatellite or allozyme analyses in the Czech Republic [29], Sweden [30] and Ukraine [16]. These hybrids were of diploid, triploid and tetraploid constitutions, respectively. Hybrids between *C*. *carassius* and *C*. *gibelio* are usually tetraploid, whereas *C*. *carassius* and *C*. *auratus* hybrids can be either diploid or triploid [16]. Mitochondrial DNA analysis showed that the *C*. *gibelio* genome was inherited from the mother [29].

Karyotypic diversity in the complex is also increased by gynogenesis, a sperm-dependent parthenogenesis that occurs when a sperm triggers the ontogenetic development of an egg typically without true fertilization [27,31]. When heterologous sperm (i.e. from another species) initiates embryonic development, newly arisen gynogenetic descendants may inherit small parts of paternal genetic material [32,33], a phenomenon called leaky gynogenesis *sensu* Janko et al. [34]. This mode of reproduction can also lead to an increase in ploidy level by a genome addition when an egg is fertilized [21,35,36]. For example, Knytl et al. [37] reported a tetraploid hybrid of *C*. *gibelio* and *C*. *carassius* (genome ratio 3 *gibelio*: 1 *carassius*) as a result of the genome addition of a haploid *C*. *carassius* chromosome complement to a triploid *C*. *gibelio* egg. The mechanism of fertilization is influenced by the mechanism of homologous/heterologous sperm recognition by the ovum [38,39]. Even the eggs of allotetraploid *Carassius* containing 212 chromosomes [40] are capable of receiving a partial chromosomal complement from homologous sperm. The resulting progeny could be hypertetraploid with 230–240 chromosomes [41].

Here we studied a reference population to Central European *C*. *carassius*. After sampling one population from a small pond in Helsinki, Finland–an area where pure *C*. *carassius* populations were expected [13]–one fish with a different ploidy level was discovered. We therefore tested, whether some polyploids and hybrids occur among pure diploid *Carassius* individuals. Specifically, we identified a triploid *Carassius* female that has an odd (3n = 156) and previously undescribed chromosome number, with a clear external morphological appearance similar to that of the European crucian carp (*C*. *carassius*). We verified whether this individual had a hybrid nature, and present two possible scenarios to explain its origin using both molecular phylogenetic and cytogenetic tools. In general, the finding of wide cryptic *Carassius* diversity should help us to protect the native, pure *C*. *carassius* in Europe.

## Material and methods

### Ethic statement

The fish were collected in accordance with the national legislation of the country concerned. All experimental procedures involving fish were approved by the Institutional Animal Care and Use Committee of the Czech Academy of Sciences, Institute of Animal Physiology and Genetics (Inst Anim Physiol & Genet), according to the directives from the State Veterinary Administration of the Czech Republic, permit number 124/2009, and by the permit number CZ 00221 issued by the Ministry of Agriculture of the Czech Republic. LC is a holder of the Certificate of Competency according to §17 of the Czech Republic Act No. 246/1992 coll. on the Protection of Animals against Cruelty (Registration number CZ 02361), provided by the Ministry of Agriculture of the Czech Republic, which authorizes animal experiments in the Czech Republic.

### Fish sampling, rearing, morphological characteristics and ploidy analyses

In total, 30 sub-adult *Carassius* individuals were collected from a small pond in Helsinki (N60.222277, E25.023085). All 30 *Carassius* individuals were identified by external morphological features given in Kottelat and Freyhof [3]; Baruš and Oliva [42]: black dot at the base of caudal peduncle, convex/concave upper edge of the dorsal fin, convex dorsal margin of head, numbers of dorsal fin rays, anal fin rays, numbers of scales in, above and below lateral line, and colour of peritoneum. The individuals were transported live to the Inst Anim Physiol & Genet and kept in 50 litres of recirculated freshwater at room temperature (22–24°C). The water was filtered and aerated; the bottom of the aquarium was covered with sand and potted water plants (genus: *Cryptocoryne*). The individuals were fed frozen mosquito larvae and fish food flakes.

**Fig 1:**
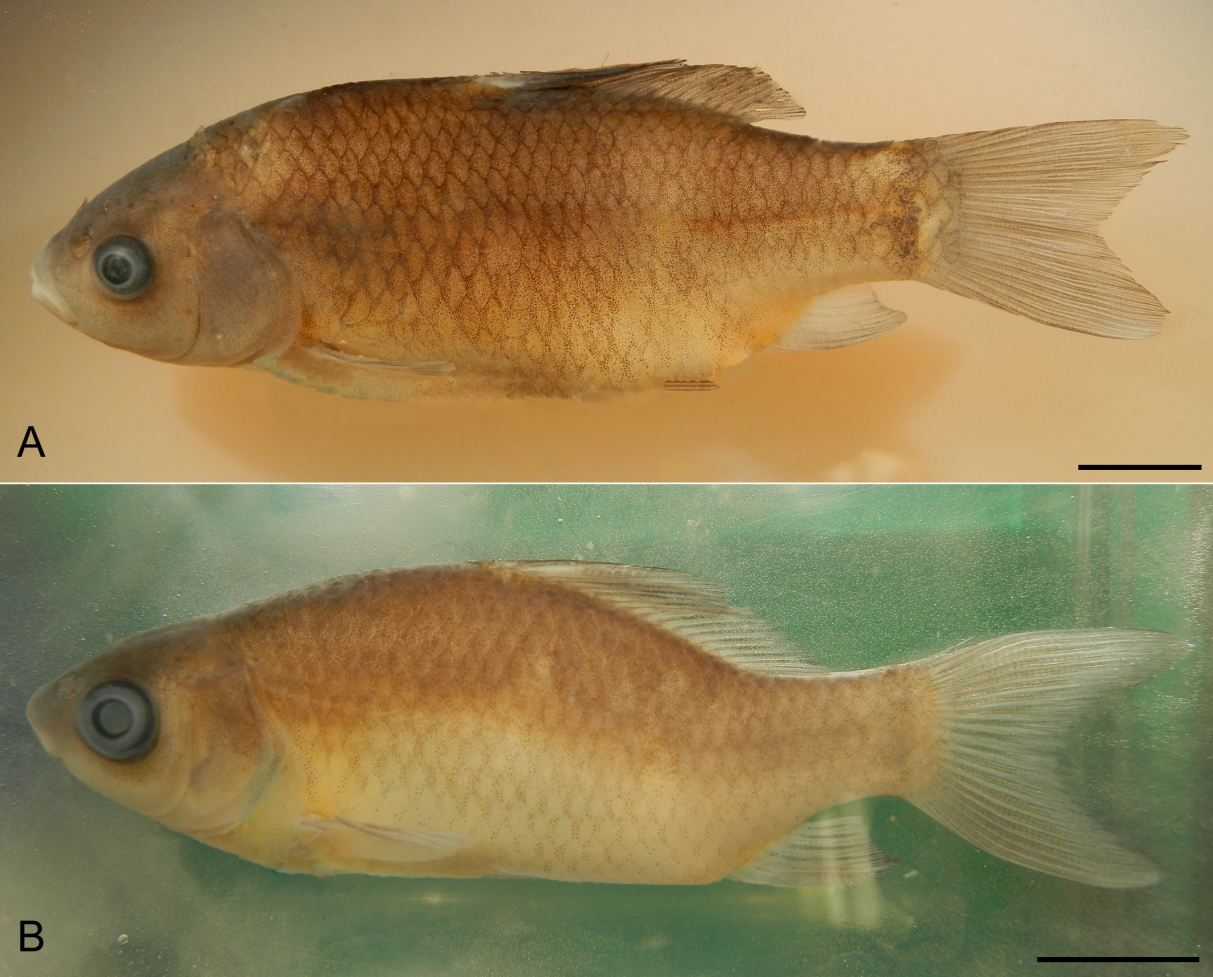
***Carassius* specimens from Helsinki, Finland**; labelled as (A) CCAHe1Fi, (B) CCAHe2Fi showing all external morphological features of *C*. *carassius*. Scale bar = 1 cm.

In order to verify ploidy levels, all individuals were analysed by flow cytometry on fin clip samples stored in 70% ethanol. Chicken blood was used as a reference standard for cell size measurement. Relative nuclear DNA content was measured with DAPI fluorochrome using the Cystain two Step High Resolution DNA Staining commercial kit (Partec GmbH, Münster, Germany). Fluorescence intensity of 5,000 stained nuclei was measured with a Partec PAII flow cytometer at a speed of 0.5 μl/s. Flow cytometric histograms were evaluated using FloMax 2.52 (Partec GmbH). A single triploid and seven randomly chosen diploid representatives were sacrificed and used for cytogenetic investigation (Fig 1). See Chromosome Preparation section for details on anaesthesia during experiments. Fin clips from a triploid and a representative diploid individual were used for molecular analyses to investigate their genotypes. These individuals were deposited as voucher specimens in the collection of the Inst Anim Physiol & Genet under the codes CCAHe1Fi and CCAHe2Fi, respectively.

### Chromosome preparation

Mitotic activity was stimulated by intraperitoneal injection of 0.1% CoCl_2_ (1 ml CoCl_2_/ 100 g body weight) into the abdominal cavity of two individuals 24 hours (h) before chromosome preparation. Standard direct procedures for chromosome preparation from the cephalic kidney followed Bertollo and Cioffi [43]. The individuals were anesthetised by incubation for 5 min in 2-Phenoxyethanol (Sigma-Aldrich, St. Louis, MO, USA). Valid Animal Use protocols were followed at the Inst Anim Physiol & Genet (16OZ25207/2014-17214, Č.j. 9321/2015-MZE-17214 and 17OZ25208/2014-17214, Č.j. 9322/2015-MZE-17214), and Czech University of Live Sciences Prague (02PP/2012) during this study. Cell suspensions were spread onto clean microscopic slides one day before use for FISH, followed by conventional chromosome banding. Chromosome slides were stored at -4°C.

### Molecular phylogenetic analyses

Genomic DNA was isolated from ethanol-preserved fin clips using DNeasy Blood and Tissue Kit (Qiagen GmbH, Hilden, Germany) according to manufacturer’s instructions. The mitochondrial cytochrome *b* gene (cyt *b*) was amplified using primers GluL (GAA CCA CCG TTG TTA TTC AA) and ThrH (ACC TCC RAT CTY CGG ATT ACA); [44]. PCR amplification was performed as described in Rylková et al. [17]. The ribosomal protein S7 was amplified using primers S7RPEX1F (TGG CCT CTT CCT TGG CCG TC) and S7RPEX2R (AAC TCG TCT GGC TTT TGC CC); [45]. The PCR reaction contained 1.5 μl of template DNA, 1.5 μl of each primer, 12.25 μl of Combi PPP Master Mix (Top-Bio, Prague, Czech Republic) and ddH_2_O up to a total volume of 25 μl. The PCR profile (carried out on an MJ Research PTC-1148 thermocycler) started with a 5 minute (min) period of initial denaturation at 95°C, followed by two cycles of: 94°C for 1 min, 60°C for 90 seconds (s) and 72°C for 2 min; two cycles of: 95°C for 1 min, 58°C for 90 s and 72°C for 2 min; two cycles of: 94°C for 1 min, 56°C for 90 s and 72°C for 2 min; 30 cycles of: 94°C for 1 min, 54°C for 90 s and 72°C for 2 min. The PCR was terminated with a final elongation step of 72°C for 7 min. In the case of the ribosomal protein S7, multiple amplicons per sampled individual were processed. All PCR products were purified and sequenced in both forward and reverse directions at Macrogen Inc., Seoul, Korea. The raw chromatograms were manually assembled and checked by eye for potential errors using BioEdit 5.0.9 software [46]. Different alleles from heterozygous individuals were separated manually. Using homozygous individuals *C*. *carassius* (KX688792, KP153224) and *C*. *gibelio* (KR054639, KR054650) as a reference with a species-specific variation, we were able to manually separate individual haplotypes from heterozygous sequences of a triploid individual.

Sequences were deposited to the GenBank database (accession numbers listed in Table 1). The phylogenetic relationships were estimated using maximum parsimony (MP) in PAUP* ver. 4.0b10 [47], as well as Bayesian analysis (BAY) using the program MrBayes ver. 3.0 [48]. The BAY analysis of mtDNA was based on estimated models (GTR+Γ) taking into consideration six rate categories and the gamma distribution of mutation rates. Starting from a random tree, two parallel runs, each consisting of six Monte Carlo Markov Chains, were run simultaneously for 1,000,000 generations with a sampling frequency of 100. For the MP analysis, statistical support was assessed using 1000 non-parametric bootstrap resamplings for each dataset, and the resulting trees were used to build 50% majority rule consensus trees in PAUP*. Both phylogenetic trees were rooted with the common carp *Cyprinus carpio* (Linnaeus, 1758) as an outgroup. Accession numbers of carp sequences are included in Table 1.

**Table 1:** List of *Carassius* samples included in molecular phylogenetic analyses.

sample	Cyt *b*	S7	Locality	origin/present status
**CCAHe1Fi**	KX688783	KR054635	Finland	hybrid
		KR054636		
		KR054637		
**CCAHe2Fi**	KR131843	KX688791	Finland	native/vulnerable
***C*. *carassius***	KR131839	KX688792	Czech Republic	native/critically endangered
***C*. *carassius***	KR131840	KX688793	Czech Republic	
***C*. *gibelio***	KX688786	KR054639	Czech Republic	introduced/established
***C*. *gibelio***	KX688785	KR054650	Germany	introduced/established
***C*. *gibelio***	KX688784	KR054641	Czech Republic	
***C*. *auratus***	KX688781	KX688787	Czech Republic	introduced/not established
***C*. *auratus***	KX688782	KX688788	Czech Republic	
		KX688789		
***C*. *auratus***	EU663576 [49]	KX688790	Czech Republic	
***C*. *langsdorfii***	DQ399921 [50]	KP153186	Japan	native/established
		KP153187		
***C*. *langsdorfii***	JN412529 [17]	KP153183	Czech Republic	introduced/not established
***C*. *langsdorfii***	GU942710 [51]	KP153182	Bosnia and Hercegovina	introduced/not established
***C*. *cuvieri***	JN402304 [52]	KP153216	Japan	native/established
		KP153217		
***Cyprinus carpio***	HM008692 [51]	KP153228	Thailand	established
		KP153229		

For cyt *b* gene and S7 gene, GenBank accession numbers are given together with a reference where appropriate. The other sequences were deposited to the GenBank database. Origin and present status were taken from the locality of collection.

### Preparation of the 5S and 28S rDNA probe and FISH

FISH with pike *Esox lucius* (Linnaeus, 1758) 5S and 28S rDNA probes were used. Genomic DNA from pike was amplified with either 5S or 28S primers (Integrated DNA Technologies), PPP Master Mix with Taq DNA polymerase (Top-Bio) using PCR reaction (50 μl total volume), and the purified PCR product was indirectly labelled by either Digoxigenin-11-dUTP or Biotin-16-dUTP (both Roche, Mannheim, Germany) by PCR reaction again. The sequences of 5S primers were designed according to Komiya and Takemura [53]: 5’-CAGGCTGGTATGGCCGTAAGC-3’ and 5’-TACGCCCGATCTCGTCCGATC -3’; and those of the 28S primers were: 5’- AAACTCTGGTGGAGGTCCGT -3’ and 5’- CTTACCAAAAGTGGCCCACTA -3’ [54]. The temperature profile for the amplification of the 5S locus was: initial denaturation step for 5 min at 95°C, followed by 34 cycles (95°C for 15 s, 55°C for 30 s and 72°C for 30 s) and a final extension step at 72°C for 5 min [55] slightly modified by [56]. Conditions for PCR of the 28S locus were as follows: initial denaturation step for 3 min at 94°C, followed by 33 cycles (94°C for 30 sec, 53°C for 30 sec and 72°C for 45 sec) with final extension step at 72°C for 10 min [57] and modified by [56]. PCR products were separated on a 0.8% agarose gel using TBE buffer. 300 ng of total DNA was precipitated with 5 μl salmon sperm (100 μg/ml), 3M sodium acetate pH = 5.2 (25°C) and 96% ethanol (-20°C), washed in 70% ethanol, dried at 37°C and re-suspended in 25 μl of hybridization buffer. The buffer contained components according to Cremer et al. [58] and Symonová et al. [59].

Chromosomes were dehydrated through ethanol series (70, 80 and 96% for 3 min each) at room temperature and air-dried, then aged overnight at 37°C one day before hybridization. Chromosomes were treated for 1 h at 60°C on a heating plate, then incubated in 10 μl DNase-free RNase (25 μg/ 1.25 ml H_2_O) with 500 μl 2X SSC, and pepsinized (following Symonová et al. [59]). After RNase treatment and pepsinization, slides were dehydrated through ethanol series (70, 80 and 96% for 3 min each) at room temperature and air-dried.

Hybridization and detection during FISH experiments were carried out as described by Cremer et al. [58] and Knytl et al. [37] with minor modifications. After dropping the hybridization mixture, the slides were incubated for 24 h (instead of 48 h) at 37°C in a dark room. A series of stringency washes was performed according to Zhu et al. [60]. The blocking reaction was carried out with 3% BSA/ 4X SSC/ 0.1% Tween. The Digoxigenin-11-dUTP/Biotin-16-dUTP labelled probe was detected by Anti-Digoxigenin-Fluorescein (Roche)/CY^TM^3-Streptavidin (Invitrogen, Camarillo, CA, USA) respectively, diluted according to manufacturer’s instructions. Chromosomes were counterstained with Vectashield/DAPI (Vector, Burlingame, CA, USA). 40 metaphase spreads with rDNA probes were analysed per individual.

### Microscopy and image processing

FISH images were captured with a cooled CCD camera Olympus DP30BW (equipped with a black-and-white [B&W] CCD-Chip Sony ICX285-AL) coupled to an epifluorescence microscope Olympus AX70 equipped with a set of three narrowband fluorescent filters. Images were processed with Olympus Acquisition and Micro Image software, respectively. Chromosome morphology was determined according to Guerra et al. [61]. Chromosomes were identified using the chromosomal nomenclature described in Knytl et al. [25,37]. In the case of poorly distinguishable chromosomes, ACC Image Analyzer (6.2) was used for determining of the *p*/*q* arm ratio. Chromosomes were arranged into karyograms using Adobe Photoshop (CS7).

### Conventional chromosome banding

After the FISH experiment the chromosome slides were cleaned in xylene for 2 min, benzoin for 2 min, then incubated in 4X SSC/ 0.1% Tween for 30 min at 44°C. Dehydration through an ethanol series on ice was then performed (70, 80 and 96% for 3 min each). After dehydration, chromosomes were destained in fixative (methanol: acetic acid; 3:1, v/v) for 30 min at room temperature, washed with distilled H_2_O and air-dried. Sequential chromosome banding (DAPI/CMA_3_, DAPI/C-banding) was carried out according to Rábová et al. [62] with destaining of slides between CMA_3_ and C-banding. 40 images for each banding type (i.e. CMA_3_, DAPI, C-banding) were analysed per individual.

## Results

### Morphological identification

Morphologically, all 30 *Carassius* individuals were identified as *C*. *carassius*. Diagnostic characters for *C*. *carassius* determination included mainly the convex upper edge of the dorsal fin and whitish peritoneum. No individual was morphologically different from the others, and no morphological features displayed hybrid morphology. Morphological characteristics of CCAHe1Fi and CCAHe2Fi individuals are shown in Table 2.

**Table 2:** Morphological characteristics of a triploid, diploid representative and *C*. *carassius*.

	CCAHe1Fi hybrid female	CCAHe2Fi male	*C*. *carassius*
**black dot at the base of caudal peduncle**	yes	yes	yes
**upper edge of the dorsal fin**	slightly convex	slightly convex	convex
**dorsal margin of head**	convex	convex	convex
**dorsal fin rays**	III 16	III 17	III–IV 14–25
**anal fin rays**	II 6	II 7	II-III 5–8
**scales in lateral line**	30	34	(30) 31–36 (37)
**scales above lateral line**	6	6	6–8
**scales below lateral line**	7	7	5–7
**peritoneum**	whitish	whitish	whitish

A triploid and a diploid *Carassius* individual from Helsinki, Finland (this study). Diagnostic characters of *C*. *carassius* followed those in Baruš and Oliva [42]; Kottelat and Freyhof [3].

### Ploidy level determination

A single *Carassius* individual (CCAHe1Fi) was identified as a triploid female, while the other 29 individuals were diploids. Cytogenetic analysis revealed 156 chromosomes in this triploid. Seven reference diploids had 100 chromosomes, including the male individual used in phylogenetic analysis (CCAHe2Fi).

### Phylogenetic identification

**Fig 2:**
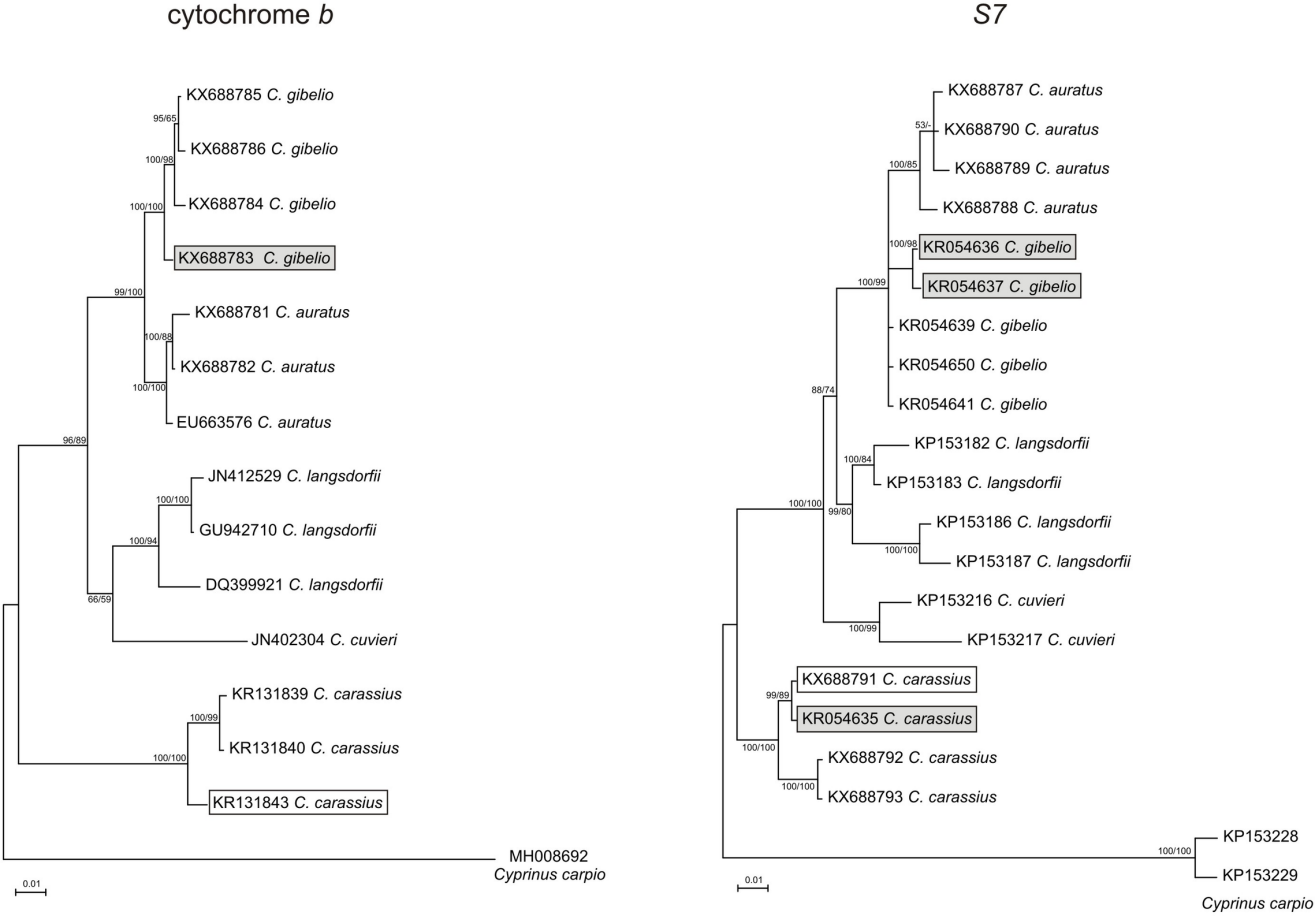
Reconstructed *Carassius* phylogeny of the mitochondrial cyt *b* and nuclear S7 sequences. Topologies of phylogenetic trees follow BAY analysis graphical outline. Numbers at the nodes represent statistical support for BAY and MP analyses, respectively. Bootstrap supports below 50 and Bayesian posterior probabilities below 0.75 are not shown. Sequences of the analysed individuals: hybrid female (CCAHe1Fi) and male (CCAHe2Fi) are highlighted by the grey and white rectangles, respectively.

The final matrix of the cyt *b* sequences of the triploid female (CCAHe1Fi) and diploid male (CCAHe2Fi) consisted of 1,114 base pairs (bps) containing 248 variable characters with 151 parsimony informative sites. In the case of the S7 sequences, the final matrix consisted of 758 bps containing 184 variable characters, of which 148 were parsimony informative. Both analytical methods recovered trees of very similar topologies with high statistical support. In both cases, the sequences formed five well-supported lineages–*C*. *auratus*, *C*. *gibelio*, *C*. *langsdorfii*, *C*. *cuvieri* (Temminck & Schlegel, 1846) and *C*. *carassius* (Fig 2).

Analyses of mitochondrial DNA identified individual CCAHe1Fi as *C*. *gibelio* and CCAHe2Fi as *C*. *carassius*. The ribosomal S7 gene gave further support that CCAHe2Fi is *C*. *carassius*.

Alleles differed in heterozygous individuals in their length mainly due to short insertions and deletions (indels), which occur in specific stretches of the sequences. These stretches were analogous in all lineages, but the total length of gained sequences differ significantly when comparing *Carassius* lineages. The *C*. *carassius* male was homozygous.

For the individual CCAHe1Fi, we reconstructed three haplotypes in the S7 gene. Two haplotypes segregated with typical *C*. *gibelio* alleles and one haplotype with *C*. *carassius* alleles, supporting a triploid constitution for CCAHe1Fi and its hybrid origin.

### Fish

**Fig 3:**
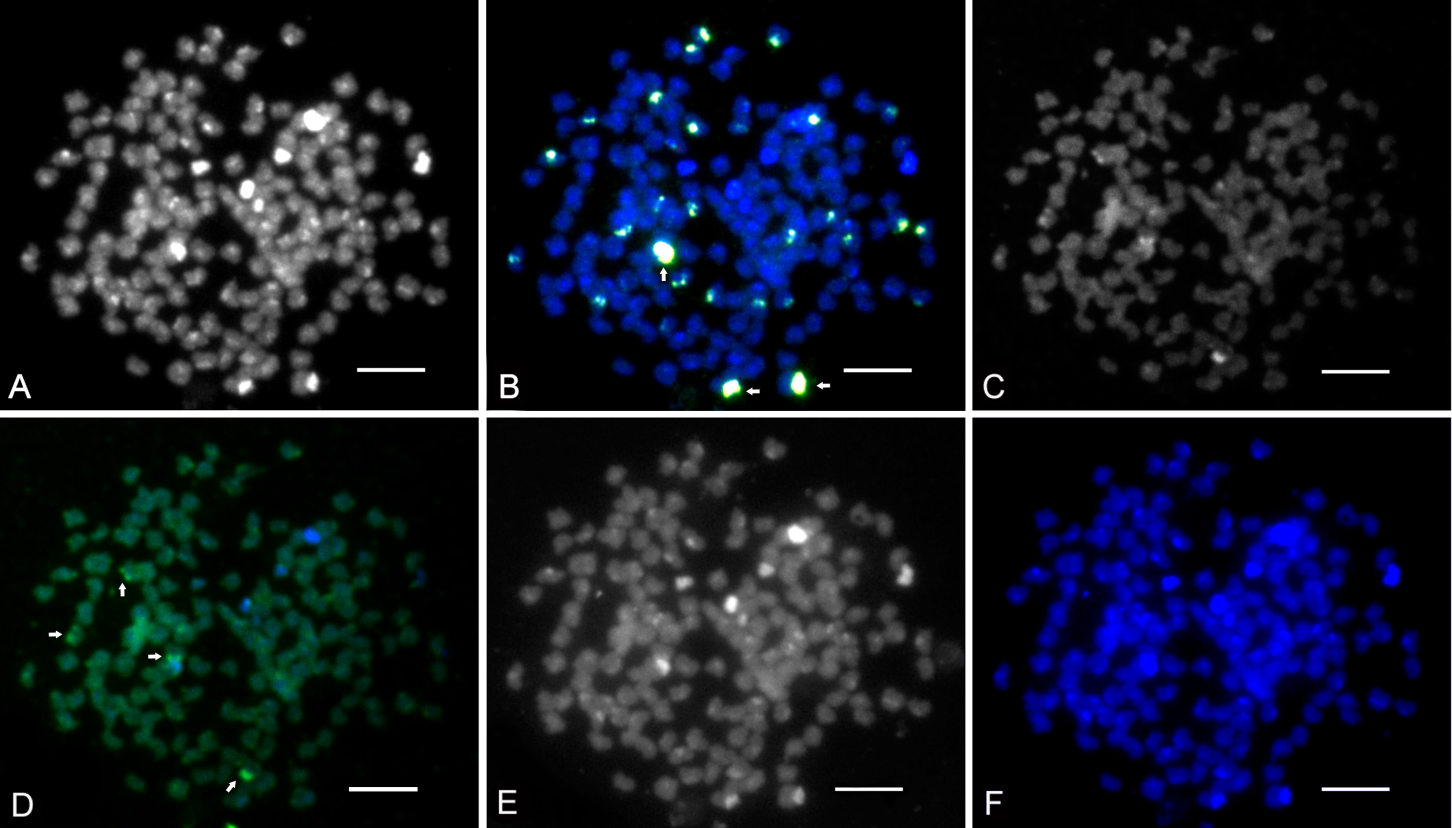
**Metaphases of hybrid CCAHe1Fi female (3n = 156),** (A) Counterstained by DAPI showed all 156 chromosomes with nine positive blocks; B&W. (B) 5S rDNA probe showing 27 loci; green, three of nine DAPI-positive blocks co-localized with 5S rDNA fragments. Three strong signals are indicated by arrows. (C) Chromosomes stained by CMA_3_ showing four NORs; B&W, (D) Pseudo-coloured with DAPI (blue), CMA_3_ (green) indicated by arrows. (E) C-banded chromosomes showing 27 heterochromatic blocks situated in the pericentromeric chromosome regions; B&W, (F) Pseudo-coloured with DAPI (blue). Scale bars = 10 μm.

**Fig 4:**
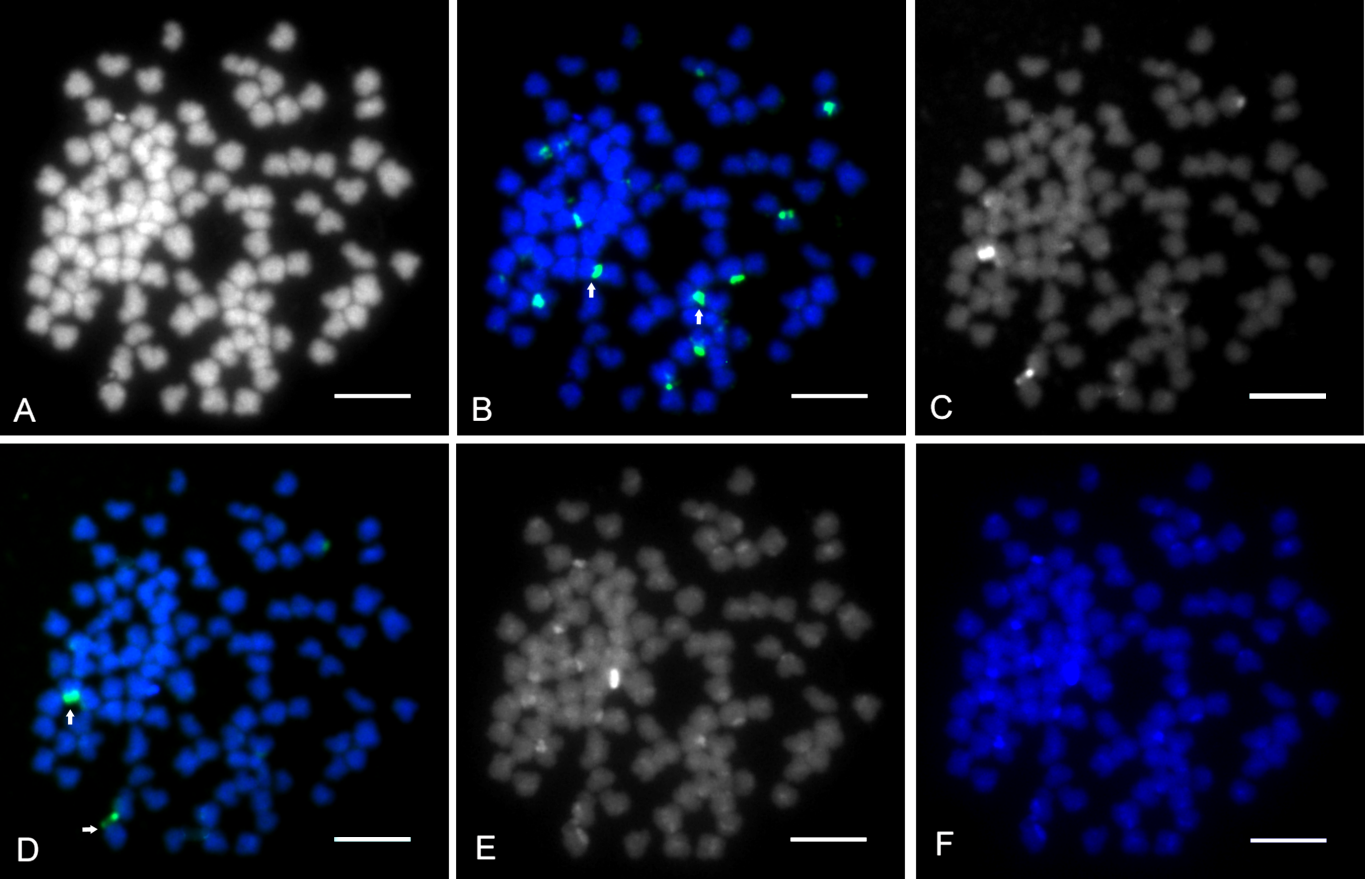
**Metaphases of diploid CCAHe2Fi male (2n = 100),** (A) Counterstained by DAPI showing consistently bright labelling of all 100 chromosomes with no positive blocks; B&W. (B) 5S rDNA probe showing 18 loci; green. Two strong signals are indicated by arrows. (C) Chromosomes stained by CMA_3_ showing two NORs; B&W, (D) Pseudo-coloured with DAPI (blue), CMA_3_ (green) indicated by arrows. (E) C-banded chromosomes showing 18 heterochromatic blocks located in the pericentromeric chromosome regions; B&W, (F) Pseudo-coloured with DAPI (blue). Scale bars = 10 μm.

**Fig 5:**
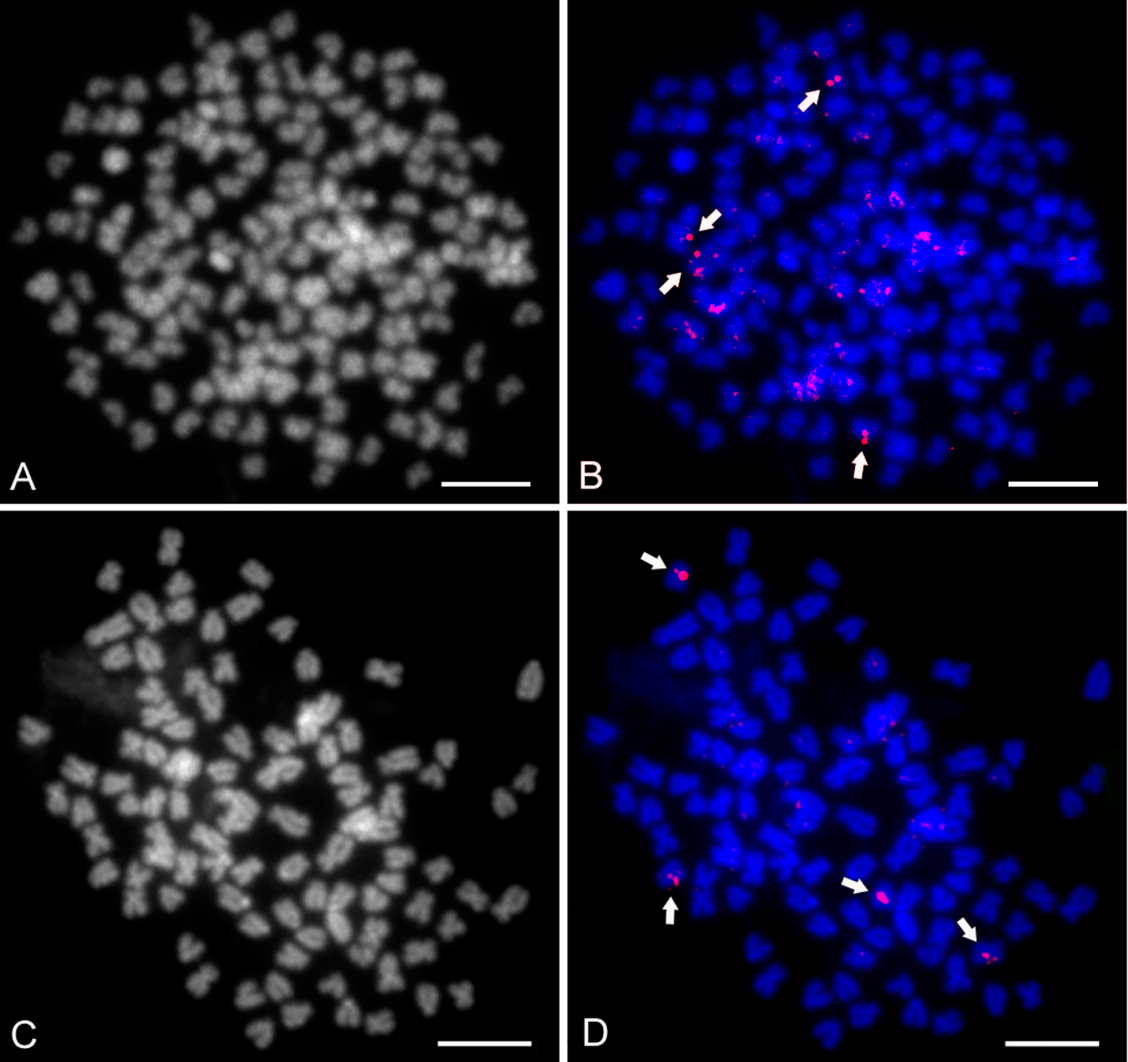
28S rDNA FISH. (A, C) Chromosomes counterstained by DAPI; B&W. 28S rDNA probe showing four signals; red, both on (B) CCAHe1Fi female and (D) CCAHe2Fi male. 28S rDNA FISH signals are indicated by arrows. Scale bars = 10 μm.

DAPI counterstained all 156 chromosomes in the CCAHe1Fi female (Fig 3A) and 100 chromosomes in the CCAHe2Fi male (Fig 4A), here representing shared cytogenetic patterns among seven karyologically investigated individuals. In the CCAHe1Fi female with 156 chromosomes, strong 5S rDNA loci were visible on the *p* arms of three *sm* chromosomes and 24 weak signals on *sm* and *st-a* chromosomes (Fig 3B). In the CCAHe2Fi male, strong 5S rDNA signals were situated on the *p* arms of two *sm* chromosomes and weak signals on 16 *sm* and *st-a* chromosomes (Fig 4B). DAPI labelled AT-rich heterochromatic blocks on nine chromosomes in the CCAHe1Fi female (Fig 3A); six bore consistently intensive fluorescent signals and three DAPI-positive chromosomes co-localized with 5S rDNA-positive sites located on the *p* arms of three *sm* chromosomes. No DAPI-positive signals were detected in chromosome sets of the CCAHe2Fi male (Fig 4A). Both in the CCAHe1Fi female and CCAHe2Fi male, 28S rDNA loci were visible on the *p* arms of two *sm* chromosomes and two *st-a* chromosomes (Fig 5).

### Karyotype analysis

**Fig 6:**
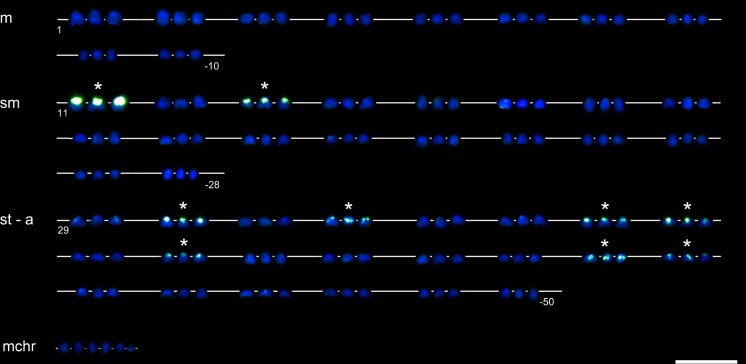
Karyotype of the CCAHe1Fi female with 156 chromosomes demonstrating 27 5S rDNA signals. Three strong on *sm*, three weak on *sm* and 21 signals on *st-a* chromosomes, DAPI (blue) and FITC filter (green). All nine triplets are highlighted by asterisks. Scale bar = 10 μm.

**Fig 7:**
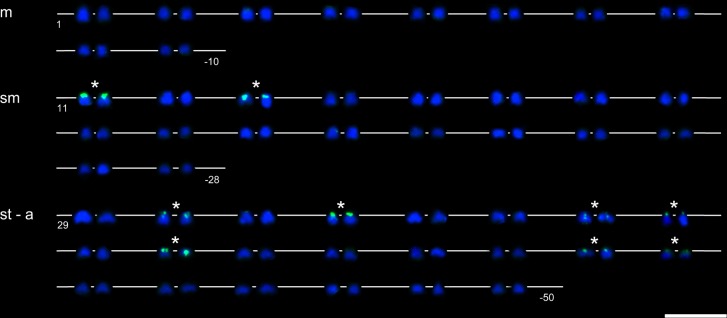
Karyotype of the CCAHe2Fi male with 100 chromosomes with 18 5S rDNA signals. Two strong on *sm*, two weak on *sm* and 14 signals on *st-a* chromosomes, DAPI (blue) and FITC filter (green). All nine chromosome pairs are highlighted by asterisks. Scale bar = 10 μm.

The CCAHe1Fi female karyotype (3n = 156) was composed of 30 metacentric (*m*), 54 submetacentric (*sm*) 66 subtelo- to acrocentric (*st-a*) and 6 microchromosomes. There were 27 loci–consistent with nine triplets of 5S rDNA-bearing chromosomes (Fig 6)–reflecting its triploidy. The representative diploid CCAHe2Fi male (2n = 100) possessed 10 pairs of *m*, 18 pairs of *sm* and 22 pairs of *st-a* chromosomes in its karyotype. In this case, 18 5S rDNA loci labelled nine homologous chromosome pairs (Fig 7). The number of chromosomes was counted under DAPI fluorescence.

### Chromosome banding

Sequential fluorescent banding (DAPI/CMA_3_, DAPI/C-banding) on destained slides after the FISH experiment revealed different numbers of signals. All CMA_3_-positive bands were situated at the sites of the secondary constrictions on the *p* arms of *sm* or *st-a* chromosomes. C-banded regions showed blocks of constitutive heterochromatin at the pericentromeric chromosome regions. The karyotype/chromosome set of triploid CCAHe1Fi female contained four to six CMA_3_-positive bands (Fig 3C and 3D) and 27 C-positive heterochromatic blocks (Fig 3E and 3F) while diploid individuals including CCAHe2Fi male contained two to four CMA_3_-positive bands (Fig 4C and 4D) and 18 C-positive heterochromatic blocks (Fig 4E and 4F). Data regarding chromosome examination is summarized in Table 3.

**Table 3:** Chromosomal characteristics of a diploid and a triploid *Carassius* representative.

individual	chromosome number	5S rDNA^+^ loci	28S rDNA^+^ loci	DAPI^+^	CMA_3_^+^	C-banding
**CCAHe1Fi**	156	3+24	4	9	4–6	27
**CCAHe2Fi**	100	2+16	4	0	2–4	18

Cytogenetic characteristic of two *Carassius* individuals from Helsinki (Finland). Differences between CCAHe1Fi and CCAHe2Fi are unambiguous in each chromosomal characteristic (except of the 28S rDNA loci number).

## Discussion

### Genetic markers unravelling hidden *Carassius* diversity

Both natural and anthropogenic introduction of invasive species, habitat loss and degradation, predation, draining of wetland pools and genetic contamination are some of the factors that may eradicate populations of native *C*. *carassius* [7,10,18,63]. Our genetic analyses contributed to cytotaxonomy of the endangered *C*. *carassius* [25,64] and revealed a haploid *C*. *carassius* genome in a triploid hybrid combined with a diploid *C*. *gibelio* genome.

A convex upper edge of the dorsal fin and whitish peritoneum were fundamental characteristics for *C*. *carassius* determination. *C*. *gibelio* and *C*. *auratus* usually have a concave upper edge of the dorsal fin and black peritoneum [3]. Despite its morphological similarity to *C*. *carassius*, the nuclear genome of the triploid *Carassius* female consisted of only one set of *C*. *carassius* chromosomes–the other two sets were that of *C*. *gibelio*. A different dosage was evident in heterozygous sites of segregating species-specific alleles, where allele peaks of *C*. *gibelio* were approximately twice as high as those of *C*. *carassius*, as similarly described in Janko et al. [34] for triploid spined loaches of hybrid origin. The cyt *b* gene placed this female in the *C*. *gibelio* mitochondrial lineage, indicating maternal origin. Thus, the genetic composition of this triploid individual is CGG^mtDNA G^ (C = *C*. *carassius*; G = *C*. *gibelio*), which suggests that in the parental generation, (i) a diploid egg (CG) was fertilised by a haploid sperm cell bearing a *C*. *gibelio* (G) genome or (ii) a diploid egg (GG) was fertilised by a haploid sperm cell (C). This type of reproduction was already described e.g. in fishes of the genus *Leuciscus*, where hybrid eggs could be fertilised by one of two parental species [65]. The capability to produce unreduced diploid gametes in *Carassius* fishes was already proved experimentally [39,66] by an intentional crossbreeding experiment. Low haplotype diversity and homozygous allele composition in the nuclear genes of *C*. *carassius* is not surprising, as same as high haplotype diversity and heterozygosity in *C*. *gibelio*. Interestingly, mitochondrial analyses show quite low genetic diversity of widely distributed *C*. *gibelio*, with only one or two dominant haplotypes regardless of origin [17]. Phylogeny of the *C*. *auratus* complex from East Asia containing diploid, triploid and tetraploid individuals suggested that triploid *Carassius* individuals have arisen from diploids that co-occur with them and *vice versa* [19].

Some questions still remain unanswered, like (i) whether hybridization is the cause of the decline of *C*. *carassius* in Europe; and (ii) whether the *Carassius* cryptic diversity represents a threat for native species. The asexual gynogenetic mode of reproduction supposedly prevails in polyploid *Carassius* hybrids and forms clonal lineages [67]. Gynogenetic reproduction gives rise to a higher number of progeny than sexual reproduction [31] because asexual gynogens are usually not limited by the searching for a sexual partner, and they generate almost all female populations [68]. From this point of view, such a polyploid hybrid female may represent a theoretical threat for pure populations.

### Pattern of FISH, CMA_3_ and C-banding markers

FISH with rDNA probes and conventional banding techniques have been frequently used for elucidating chromosomal evolution in general, and more specifically with phenomena associated with genome duplication by allopolyploidization [69]. A specific phenomenon represents a number of strong 5S rDNA-bearing chromosomes in the triploid and diploid biotypes of *Carassius*. Three strong 5S rDNA signals verified the triploid origin of the hybrid female. A similar morphology of rDNA-positive triplets in the hybrid *Carassius* female indicated homoeologous chromosome groups in its karyotype, which resulted from a polyploidization process suggestive of hybridization [59]. Zhu et al. [60] used 5S rDNA FISH and chromosome painting probes in the chromosomes of *C*. *gibelio* (3n = 162) and *C*. *auratus* (2n = 100). They found three strong and six to 18 weak 5S rDNA-positive sites in the genome of *C*. *gibelio*; *C*. *auratus* possessed two strong and two to eight weak 5S rDNA-positive sites supporting their triploid and diploid status, respectively [60]. In *C*. *carassius* with 100 chromosomes, Spoz et al. [64] found eight to 14 5S rDNA loci on the *p* arms of *sm* chromosomes, and on the *p* arms or in a pericentromeric position of *sm* and *st-a* chromosomes. The most frequent number of 5S rDNA loci was 10, six of which gave strong signals and four gave weak signals. Diploid individuals of *Cyprinus carpio* had four to eight 5S rDNA signals [70], while the diploid *C*. *carassius* male from this study had two strong and 16 weak 5S rDNA signals. Although variation in the number of 5S rDNA signals can be found in diploids (e.g. *Carassius*, *Cyprinus*), the number of strong 5S rDNA signals is nevertheless a suitable marker to recognize polyploidization events within the genus *Carassius* [60]. 28S rDNA genes, which make up the 45S rDNA (nucleolar organizer regions [NOR]-bearing) transcriptional unit, tandemly repeated with high copy numbers are useful markers for cytotaxonomic identification [71]. In accordance with Spoz et al. [64], we found four 28S rDNA loci on a pair of *sm* and *st-a* chromosomes in both diploid male and triploid *Carassius* female. This pattern of four 28S rDNA loci and its identical chromosomal positioning (this study and [64]) shows that this region of the genome is highly conserved in *C*. *carassius*. Six 45S rDNA loci were found in the allotetraploid hybrid of *C*. *gibelio* and *C*. *carpio* (4n = 212), five of which originated from the maternal *C*. *gibelio* genome (3n = 162) and one from the paternal *C*. *carpio* genome (2n = 100) [36]. Different numbers of 45S rDNA loci within individuals of the same ploidy level (*C*. *carassius–*this study x *C*. *carpio* [36]; *C*. *gibelio* [36] x hybrid of *C*. *gibelio* and *C*. *carassius–*this study) shows interspecific and intergeneric variability. With the exception of the genus *Carassius*, intra- and inter-individual variability of 45S rDNA chromosomal localization was revealed. This is unlike the stability of 5S rDNA localization in fish genera such as *Astyanax* and *Squalius* [72,73]. On the other hand, 72% of bony fishes (Teleostei) comprise 45S rDNA sequences on a single chromosome pair (reviewed in [69]).

DAPI-positive blocks were detected on the chromosomes of the triploid *Carassius* female (this study); these same regions were observed both in diploid loaches (e.g. *Schistura*, *Barbatula*) [56] and in diploid killifish *Chromaphyosemion* [74]. Our results give the first information about DAPI-positive blocks on *Carassius* chromosomes (even DAPI covers entire chromosomes) that seems to be a powerful marker for identification of polyploid *Carassius* genomes.

CMA_3_ revealed variability in the number of signals on the chromosomes of *Carassius* (two to four in diploid, four to six in triploid). The marker does not seem to be useful for ploidy identification, but it may reveal a number of CG-rich regions not associated with active NORs on chromosomes [56]. This challenges whether CMA_3_ is a useful marker for NOR identification in *Carassius*. For example, most CMA_3_- and Ag-positive sites do not correspond to strong 28S rDNA sites in an Iberian cyprinid of the genus *Squalius* [75]. Nucleolar secondary constrictions containing NORs in African clawed frogs *Xenopus* are detectable by Ag staining [76] but not by CMA_3_ [77]. Variability of CMA_3_-positive bands depends on transcriptional activity of ribosomal genes during the interphase [64]. Variability of NOR numbers might be caused by mutability of NORs, especially by translocation [78].

C-banding detected transcriptionally inactive heterochromatin, which co-localized with all DAPI-positive blocks, CMA_3_-positive bands and some of 5S rDNA signals. Knytl et al. [25] found blocks of telomeric heterochromatin on seven chromosome pairs in diploid *C*. *carassius*, and proposed that the observed pattern is species-specific. One main difference between the previous and current study lies in the focus on intraspecific variability in the number of C-positive heterochromatic blocks.

### Origin of the triploid *Carassius* female and a threat for *C*. *carassius*

**Fig 8:**
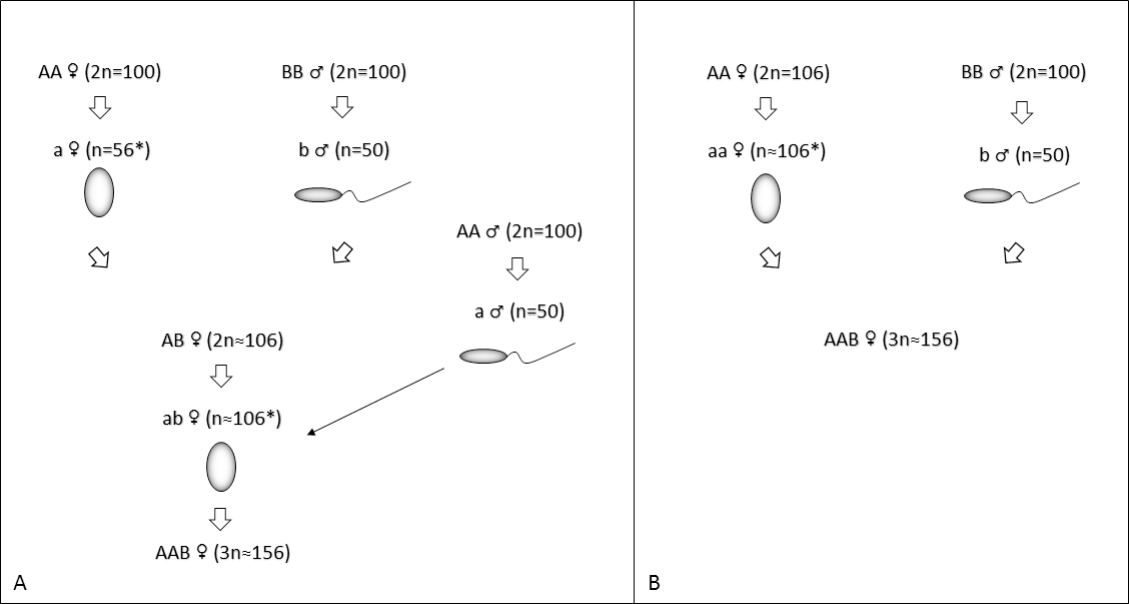
Alternative hypothetical scenarios for the origin of a triploid hybrid female with 156 chromosomes, together with the genomes of *C*. *gibelio* (A) and *C*. *carassius* (B). Scenario A) Genome addition hypothesis. Scenario B) Spontaneous allotriploid origin. Capital letters “A” and “B” denote somatic genome; lowercase letters “a” and “b” show gamete chromosome complement (both egg and spermatozoa). Asterisk marks unusual number of chromosomes in the egg. 56/106 chromosomes instead of 50/100 in female gametes, respectively, might be caused by unequal chromosome segregation during meiosis II.

There are several recognized pathways for allopolyploidy to originate among animals [79]. Combining molecular and cytogenetic data with previous knowledge on the *C*. *auratus* complex [37], we can postulate two alternative scenarios of how this particular triploid female with 156 chromosomes originated (see also Fig 8):

A) Genome addition hypothesis: Diploid *C*. *carassius* male fertilized a haploid egg of diploid *C*. *gibelio* female. A resulting hybrid progeny with 106 chromosomes produced an unreduced egg that was subsequently fertilized by a haploid sperm from *C*. *gibelio*.
B) Spontaneous allotriploid origin: *C*. *carassius* haploid sperm resulting in triploid constitution fertilized an unreduced *C*. *gibelio* egg with 106 chromosomes.

Present data do not allow us to distinguish between the two scenarios; more information about reproduction is necessary for identifying the historical scenario. The evolution of a triploid 3n = 156 remains a puzzle as well, because the chromosome number should be 3n = 150. Exceptional cases of hybrids between a *C*. *gibelio* female and *C*. *carassius* male with 102–104 chromosomes have been earlier reported, indicating that diploid stadium with increased numbers of chromosomes may be formed [21]. Unequal distribution of genetic material during meiosis, followed by the polar body extrusion might lead to the origin of hybrids even with 106 chromosomes (Fig 8). Increase of ploidy level up to 156 chromosomes might arise by addition of a haploid chromosome complement from a sperm genome to an unreduced egg. This sperm genome addition mechanism has been shown to be the origin of the allotetraploid *Carassius* [37].

This study shows that much of the European *Carassius* diversity is still unrevealed and that cryptic forms may persist undetected. It is currently difficult to estimate whether the triploid discovered in Finland represented an invasion of the *C*. *carassius* genome into *C*. *gibelio* or *vice versa*. However, the dynamics of reproductive modes in the *Carassius* complex is high. Moreover, recent declines of *C*. *carassius* across much of Europe are occurring at an alarming rate [7,80,81], and the invasive potential of *C*. *gibelio* is strong (e.g. [18]). Genetic investigations are important for conservation management for distinguishing pure *C*. *carassius* individuals from other *Carassius* hybrids, especially in the case of shared external morphological characters. Therefore, more effort in genetic investigations of *Carassius* cryptic diversity should accompany the conservation of *C*. *carassius* in Europe. Further detailed studies of the introgressive potential of such hybrids may also help to stabilize threatened populations of *C*. *carassius* in its native range.

## References

[1] Baack EJ, Rieseberg LH. A genomic view of introgression and hybrid speciation. Curr Opin Genet Dev. 2007;17: 513–518. doi: 10.1016/j.gde.2007.09.001 17933508 10.1016/j.gde.2007.09.001 PMC2173880

[2] Payseur BA, Rieseberg LH. A genomic perspective on hybridization and speciation. Mol Ecol. 2016;25: 2337–2360. doi: 10.1111/mec.13557 26836441 10.1111/mec.13557 PMC4915564

[3] Kottelat M, Freyhof J. Handbook of European freshwater fishes Cornol: Kottelat and Berlin: Freyhof; 2007.

[4] Roberts DG, Gray CA, West RJ, Ayre DJ. Evolutionary impacts of hybridization and interspecific gene flow on an obligately estuarine fish. J Evol Biol. 2009;22: 27–35. doi: 10.1111/j.1420-9101.2008.01620.x 18800995 10.1111/j.1420-9101.2008.01620.x

[5] Haynes GD, Gongora J, Gilligan DM, Grewe P, Moran C, Nicholas FW. Cryptic hybridization and introgression between invasive Cyprinid species *Cyprinus carpio* and *Carassius auratus* in Australia: implications for invasive species management. Anim Conserv. 2012;15: 83–94. doi: 10.1111/j.1469-1795.2011.00490.x

[6] Wyatt PMW, Pitts CS, Butlin RK. A molecular approach to detect hybridization between bream *Abramis brama*, roach *Rutlius rutilus* and rudd *Scardinius erythrophthalmus*. J Fish Biol. 2006;69: 52–71. doi: 10.1111/j.1095-8649.2006.01104.x

[7] Sayer CD, Copp GH, Emson D, Godard MJ, Zięba G, Wesley KJ. Towards the conservation of crucian carp *Carassius carassius*: understanding the extent and causes of decline within part of its native English range. J Fish Biol. 2011;79: 1608–24. doi: 10.1111/j.1095-8649.2011.03059.x 22136242 10.1111/j.1095-8649.2011.03059.x

[8] Mandrak NE, Cudmore B. The fall of native fishes and the rise of non-native fishes in the Great Lakes Basin. Aquat Ecosyst Health Manag. 2010;13: 255–268. doi: 10.1080/14634988.2010.507150

[9] Cambray JA. Impact on indigenous species biodiversity caused by the globalisation of alien recreational freshwater fisheries. Hydrobiologia. 2003;500: 217–230. doi: 10.1023/A:1024648719995

[10] Helfman GS. Fish conservation: a guide to understanding and restoring global aquatic biodiversity and fishery resources Washington: Island Press; 2007.

[11] Savini D, Occhipinti-Ambrogi A, Marchini A, Tricarico E, Gherardi F, Olenin S, et al The top 27 animal alien species introduced into Europe for aquaculture and related activities. J Appl Ichthyol. 2010;26: 1–7. doi: 10.1111/j.1439-0426.2010.01503.x

[12] Yang L, Sado T, Vincent Hirt M, Pasco-Viel E, Arunachalam M, Li J, et al Phylogeny and polyploidy: resolving the classification of cyprinine fishes (Teleostei: Cypriniformes). Mol Phylogenet Evol. Elsevier Inc.; 2015;85: 97–116. doi: 10.1016/j.ympev.2015.01.014 25698355 10.1016/j.ympev.2015.01.014

[13] Jeffries DL, Copp GH, Lawson Handley L, Olsén KH, Sayer CD, Hänfling B. Comparing RADseq and microsatellites to infer complex phylogeographic patterns, an empirical perspective in the Crucian carp, *Carassius carassius*, L. Mol Ecol. 2016;25: 2997–3018. doi: 10.1111/mec.13613 26971882 10.1111/mec.13613

[14] Jeffries DL, Copp GH, Maes GE, Lawson Handley L, Sayer CD, Hänfling B. Genetic evidence challenges the native status of a threatened freshwater fish (*Carassius carassius*) in England. Ecol Evol. 2017;7: 2871–2882. doi: 10.1002/ece3.2831 28479988 10.1002/ece3.2831 PMC5415527

[15] Lusk S, Hanel L, Lojkásek B, Lusková V, Muška M. The Red List of lampreys and fishes of the Czech Republic In: Němec M, Chobot K, editors. Red List of threatened species of the Czech Republic, Vertebrates. Prague: Příroda; 2017 pp. 51–82.

[16] Mezhzherin SV., Kokodii SV., Kulish AV., Verlatii DB, Fedorenko LV. Hybridization of crucian carp *Carassius carassius* (Linnaeus, 1758) in Ukrainian reservoirs and the genetic structure of hybridsHybridization of crucian carp *Carassius carassius* (Linnaeus, 1758) in Ukrainian reservoirs and the genetic structure of hybrids. Cytol Genet. 2012;46: 28–35. doi: 10.3103/S0095452712010069 22420218

[17] Rylková K, Kalous L, Bohlen J, Lamatsch DK, Petrtýl M. Phylogeny and biogeographic history of the cyprinid fish genus *Carassius* (Teleostei: Cyprinidae) with focus on natural and anthropogenic arrivals in Europe. Aquaculture. 2013;380–383: 13–20. doi: 10.1016/j.aquaculture.2012.11.027

[18] Lusková V, Lusk S, Halačka K, Vetešník L. *Carassius auratus gibelio*—The most successful invasive fish in waters of the Czech Republic. Russ J Biol Invasions. 2010;1: 176–180. doi: 10.1134/S2075111710030069

[19] Takada M, Tachihara K, Kon T, Yamamoto G, Iguchi K, Miya M, et al Biogeography and evolution of the *Carassius auratus*-complex in East Asia. BMC Evol Biol. 2010;10: 7 doi: 10.1186/1471-2148-10-7 20064277 10.1186/1471-2148-10-7 PMC2820001

[20] Ojima Y, Takai A. Further cytogenetical studies on the origin of the gold-fish. Proc Japan Acad Ser B Phys Biol Sci. 1979;55: 346–350. doi: 10.2183/pjab.55.346

[21] Toth B, Varkonyi E, Hidas A, Edvine Meleg E, Varadi L. Genetic analysis of offspring from intra- and interspecific crosses of *Carassius auratus gibelio* by chromosome and RAPD analysis. J Fish Biol. 2005;66: 784–797. doi: 10.1111/j.1095-8649.2005.00644.x

[22] Ráb P, Bohlen J, Rábová M, Flajshans M, Kalous L. Cytogenetics as a tool box in fish conservation: the present situation in Europe In: Pisano E, Ozouf-Costaz C, Foresti F, Kapoor BG, editors. Fish Cytogenetics. Enfield: CRC Press; 2007 pp. 229–241.

[23] Kalous L, Knytl M. Karyotype diversity of the offspring resulting from reproduction experiment between diploid male and triploid female of silver Prussian carp, *Carassius gibelio* (Cyprinidae, Actinopterygii). Folia Zool. 2011;60: 115–121.

[24] Boroń A, Szlachciak J, Juchno D, Grabowska A, Jagusztyn B, Porycka K. Karyotype, morphology, and reproduction ability of the Prussian carp, *Carassius gibelio* (Actinopterygii: Cypriniformes: Cyprinidae), from unisexual and bisexual populations in Poland. Acta Ichthyol Piscat. 2011;41: 19–28. doi: 10.3750/AIP2011.41.1.04

[25] Knytl M, Kalous L, Ráb P. Karyotype and chromosome banding of endangered crucian carp, *Carassius carassius* (Linnaeus, 1758) (Teleostei, Cyprinidae). Comp Cytogenet. 2013;7: 205–15. doi: 10.3897/CompCytogen.v7i3.5411 24260701 10.3897/CompCytogen.v7i3.5411 PMC3833740

[26] Cherfas NB. Natural triploidy in females of the unisexual form of silver crucian carp (*Carassius auratus gibelio* Bloch). Genetika. 1966;2: 16–24.

[27] Peňáz M, Prokeš M, Ráb P. Cytological analysis, gynogenesis and early development of *Carassius auratus gibelio*. Acta Sci Nat Brno. 1979;13: 1–33.

[28] Hänfling B, Bolton P, Harley M, Carvalho GR, Hanfling B, Bolton P, et al A molecular approach to detect hybridisation between crucian carp (*Carassius carassius*) and non-indigenous carp species (*Carassius* spp. and *Cyprinus carpio*). Freshw Biol. 2005;50: 403–417. doi: 10.1111/j.1365-2427.2004.01330.x

[29] Papoušek I, Vetešník L, Halačka K, Lusková V, Humpl M, Mendel J, et al Identification of natural hybrids of gibel carp *Carassius auratus gibelio* (Bloch) and crucian carp *Carassius carassius* (L.) from lower Dyje River floodplain (Czech Republic). J Fish Biol. 2008;72: 1230–1235. doi: 10.1111/j.1095-8649.2007.01783.x

[30] Wouters J, Janson S, Lusková V, Olsén KH. Molecular identification of hybrids of the invasive gibel carp *Carassius auratus gibelio* and crucian carp *Carassius carassius* in Swedish waters. J Fish Biol. 2012;80: 2595–604. doi: 10.1111/j.1095-8649.2012.03312.x 22650435 10.1111/j.1095-8649.2012.03312.x

[31] Lamatsch DK, Stöck M. Sperm-dependent parthenogenesis and hybridogenesis in teleost fishes In: Schön I, Martens K, Dijk P, editors. Lost Sex. Dordrecht: Springer Netherlands; 2009 pp. 399–432. doi: 10.1007/978-90-481-2770-2_19

[32] Jiang Y, Yu H, Chen B, Liang S, Shan S. Biological effect of heterologous sperm on gynogenetic offspring in *Carassius auratus gibelio*. Acta Hydrobiol Sin. 1983;

[33] Gui JF, Liang SC, Zhu LF, Jiang YG. Discovery of multiple tetraploids in artificially propagated populations of allogynogenetic silver crucian carp and their breeding potentialities. Chinese Sci Bull. 1993;38: 327.

[34] Janko K, Bohlen J, Lamatsch DK, Flajšhans M, Epplen JT, Ráb P, et al The gynogenetic reproduction of diploid and triploid hybrid spined loaches (*Cobitis*: Teleostei), and their ability to establish successful clonal lineages—on the evolution of polyploidy in asexual vertebrates. Genetica. 2007;131: 185–194. doi: 10.1007/s10709-006-9130-5 17216551 10.1007/s10709-006-9130-5

[35] Zhao J, Liu LG, Chen XL, Qing N, Dong CW. Karyotypic analysis of the multiple tetraploid allogynogenetic pengze crucian carp and its parents. Aquaculture. 2004;237: 117–129. doi: 10.1016/j.aquaculture.2004.05.001

[36] Zhu HP, Gui JF. Identification of genome organization in the unusual allotetraploid form of *Carassius auratus gibelio*. Aquaculture. 2007;265: 109–117. doi: 10.1016/j.aquaculture.2006.10.026

[37] Knytl M, Kalous L, Symonová R, Rylková K, Ráb P. Chromosome studies of European cyprinid fishes: cross-species painting reveals natural allotetraploid origin of a *Carassius* female with 206 chromosomes. Cytogenet Genome Res. 2013;139: 276–83. doi: 10.1159/000350689 23652770 10.1159/000350689

[38] Gui JF, Zhou L. Genetic basis and breeding application of clonal diversity and dual reproduction modes in polyploid *Carassius auratus gibelio*. Sci China Life Sci. 2010;53: 409–15. doi: 10.1007/s11427-010-0092-6 20596906 10.1007/s11427-010-0092-6

[39] Xiao J, Zou T, Chen Y, Chen L, Liu S, Tao M, et al Coexistence of diploid, triploid and tetraploid crucian carp (*Carassius auratus*) in natural waters. BMC Genet. 2011;12: 20 doi: 10.1186/1471-2156-12-20 21276259 10.1186/1471-2156-12-20 PMC3040159

[40] Gui J, Liang S, Zhu L, Jiang Y. Discovery and breeding potential of compound tetraploid allogynogenetic silver crucian carp in artificial population. Chin Sci Bull. 1992;37: 255–262.

[41] Gui JF, Liang SC, Zhu LF, Jiang YG. Discovery of two different reproductive development modes of the eggs of artificial multiple tetraploid allogynogenetic Silver crucian carp. Chinese Sci Bull. 1993;38: 332–337.

[42] Baruš V, Oliva O. Mihulovci (Petromyzontes) a ryby (Osteichthyes). Prague: Academia; 1995.

[43] Bertollo L, Cioffi M. Direct chromosome preparation from freshwater teleost fishes In: Ozouf-Costaz C, Pisano E, Foresti F, Foresti L de AT, editors. Fish cytogenetic techniques: Ray-Fin fishes and chondrichthyans. Enfield: CRC Press; 2015 pp. 21–26.

[44] Šlechtová V, Bohlen J, Freyhof J, Ráb P. Molecular phylogeny of the Southeast Asian freshwater fish family Botiidae (Teleostei: Cobitoidea) and the origin of polyploidy in their evolution. Mol Phylogenet Evol. 2006;39: 529–541. doi: 10.1016/j.ympev.2005.09.018 16337410 10.1016/j.ympev.2005.09.018

[45] Chow S, Hazama K. Universal PCR primers for S7 ribosomal protein gene introns in fish. Mol Ecol. 1998;7: 1255–6. doi: 10.1046/j.1365-294x.1998.00406.x 9734083

[46] Hall T. A. BioEdit: a user-friendly biological sequence alignment editor and analysis program for Windows 95/98/NT. Nucleic Acids Symp Ser. 1999;41: 95–98.

[47] Swofford D. PAUP. Phylogenetic analysis using parsimony (and other methods) Sunderland: Sinauer Associates; 2000.

[48] Huelsenbeck JP, Ronquist F. MRBAYES: Bayesian inference of phylogenetic trees. Bioinformatics. 2001;17: 754–755. doi: 10.1093/bioinformatics/17.8.754 11524383 10.1093/bioinformatics/17.8.754

[49] Rylková K, Kalous L, Šlechtová V, Bohlen J. Many branches, one root: First evidence for a monophyly of the morphologically highly diverse goldfish (*Carassius auratus*). Aquaculture. 2010;302: 36–41. doi: 10.1016/j.aquaculture.2010.02.003

[50] Kalous L, Šlechtová V, Bohlen J, Petrtýl M, Švátora M. First European record of *Carassius langsdorfii* from the Elbe basin. J Fish Biol. 2007;70: 132–138. doi: 10.1111/j.1095-8649.2006.01290.x

[51] Kalous L, Rylková K, Bohlen J, Šanda R, Petrtýl M. New mtDNA data reveal a wide distribution of the Japanese ginbuna *Carassius langsdorfii* in Europe. J Fish Biol. 2013;82: 703–707. doi: 10.1111/j.1095-8649.2012.03492.x 23398078 10.1111/j.1095-8649.2012.03492.x

[52] Kalous L, Bohlen J, Rylková K, Petrtýl M. Hidden diversity within the Prussian carp and designation of a neotype for *Carassius gibelio* (Teleostei: Cyprinidae). Ichthyol Explor Freshwaters. 2012;23: 11–18.

[53] Komiya H, Takemura S. Nucleotide sequence of 5S ribosomal RNA from rainbow trout (*Salmo gairdnerii*) liver. J Biochem. 1979;86: 1067–80. doi: 10.1093/oxfordjournals.jbchem.a132601 115850 10.1093/oxfordjournals.jbchem.a132601

[54] Naito E, Dewa K, Ymanouchi H, Kominami R. Ribosomal ribonucleic acid (rRNA) gene typing for species identification. J Forensic Sci. ASTM International; 1992;37: 396–403. doi: 10.1520/JFS13249J 1500890

[55] Alves-Costa FA, Martins C, de Matos FDC, Foresti F, Oliveira C, Wasko AP. 5S rDNA characterization in twelve Sciaenidae fish species (Teleostei, Perciformes): Depicting gene diversity and molecular markers. Genet Mol Biol. 2008;31: 303–307. doi: 10.1590/S1415-47572008000200025

[56] Sember A, Bohlen J, Šlechtová V, Altmanová M, Symonová R, Ráb P. Karyotype differentiation in 19 species of river loach fishes (Nemacheilidae, Teleostei): extensive variability associated with rDNA and heterochromatin distribution and its phylogenetic and ecological interpretation. BMC Evol Biol. 2015;15: 1–22. doi: 10.1186/s12862-014-0274-0 26573692 10.1186/s12862-015-0532-9 PMC4647339

[57] Zhang Q, Cooper RK, Tiersch TR. Chromosomal location of the 28S ribosomal RNA gene of channel catfish by in situ polymerase chain reaction. J Fish Biol. 2000;56: 388–397. doi: 10.1006/jfbi.1999.1164

[58] Cremer M, Grasser F, Lanctôt C, Müller S, Neusser M, Zinner R, et al Multicolor 3D fluorescence in situ hybridization for imaging interphase chromosomes. Methods Mol Biol. 2008;463: 205–39. doi: 10.1007/978-1-59745-406-3_15 18951171 10.1007/978-1-59745-406-3_15

[59] Symonová R, Sember A, Majtánová Z, Ráb P. Characterization of fish genomes by GISH and CGH In: Ozouf-Costaz C, Pisano E, Foresti F, Foresti L de AT, editors. Fish cytogenetic techniques: Ray-Fin fishes and chondrichthyans. Enfield: CRC Press; 2015 pp. 118–131.

[60] Zhu HP, Ma DM, Gui JF. Triploid origin of the gibel carp as revealed by 5S rDNA localization and chromosome painting. Chromosom Res. 2006;14: 767–776. doi: 10.1007/s10577-006-1083-0 17115331 10.1007/s10577-006-1083-0

[61] Guerra MDS, Valim-Labres ME, Porto MDM, Matsumura ATS. Reviewing the chromosome nomenclature of Levan et al. Brazilian J Genet. 1986;IX: 741–743.

[62] Rábová M, Völker M, Pelikánová Š, Ráb P. Sequential chromosome banding in fishes In: Ozouf-Costaz C, Pisano E, Foresti F, Foresti L de AT, editors. Fish cytogenetic techniques: Ray-Fin fishes and chondrichthyans. Enfield: CRC Press; 2015 pp. 92–102.

[63] Wheeler A. Status of the crucian carp, *Carassius carassius* (L.), in the UK. Fish Manag Ecol. 2000;7: 315–322. doi: 10.1046/j.1365-2400.2000.007004315.x

[64] Spoz A, Boron A, Porycka K, Karolewska M, Ito D, Abe S, et al Molecular cytogenetic analysis of the crucian carp, *Carassius carassius* (Linnaeus, 1758) (Teleostei, Cyprinidae), using chromosome staining and fluorescence in situ hybridisation with rDNA probes. Comp Cytogenet. 2014;8: 233–248. doi: 10.3897/CompCytogen.v8i3.7718 25349674 10.3897/CompCytogen.v8i3.7718 PMC4205492

[65] Alves MJ, Coelho MM, Collares-Pereira MJ. Evolution in action through hybridisation and polyploidy in an Iberian freshwater fish: a genetic review. Genetica. 2001;111: 375–85. 11841181 10.1023/a:1013783029921

[66] Zhang C, Liu S, Li T, Liu Y. Studies of chromosome sets in embryonic cell of hybrid fish of red crucian crap (♀)× common crap (♂). J Fish China. 2011; 1370–1373.

[67] Šimková A, Hyršl P, Halačka K, Vetešník L. Physiological and condition-related traits in the gynogenetic-sexual *Carassius auratus* complex: different investments promoting the coexistence of two reproductive forms? BMC Evol Biol. 2015;15: 154 doi: 10.1186/s12862-015-0438-6 26245328 10.1186/s12862-015-0438-6 PMC4545816

[68] Avise JC. Evolutionary perspectives on clonal reproduction in vertebrate animals. Proc Natl Acad Sci U S A. 2015;112: 8867–73. doi: 10.1073/pnas.1501820112 26195735 10.1073/pnas.1501820112 PMC4517198

[69] Gornung E. Twenty years of physical mapping of major ribosomal RNA genes across the teleosts: A review of research. Cytogenet Genome Res. 2013;141: 90–102. doi: 10.1159/000354832 24080951 10.1159/000354832

[70] Inafuku J, Nabeyama M, Kikuma Y, Saitoh J, Kubota S, Kohno SI. Chromosomal location and nucleotide sequences of 5S ribosomal DNA of two cyprinid species (Osteichthyes, Pisces). Chromosom Res. 2000;8: 193–199. doi: 10.1023/A:1009292610618 10.1023/a:1009292610618 10841046

[71] Symonová R, Majtánová Z, Sember A, Staaks GBO, Bohlen J, Freyhof J, et al Genome differentiation in a species pair of coregonine fishes: an extremely rapid speciation driven by stress-activated retrotransposons mediating extensive ribosomal DNA multiplications. BMC Evol Biol. 2013;13: 42 doi: 10.1186/1471-2148-13-42 23410024 10.1186/1471-2148-13-42 PMC3585787

[72] Gromicho M, Coutanceau J-P, Ozouf-Costaz C, Collares-Pereira MJ. Contrast between extensive variation of 28S rDNA and stability of 5S rDNA and telomeric repeats in the diploid-polyploid *Squalius alburnoides* complex and in its maternal ancestor *Squalius pyrenaicus* (Teleostei, Cyprinidae). Chromosome Res. 2006;14: 297–306. doi: 10.1007/s10577-006-1047-4 16628500 10.1007/s10577-006-1047-4

[73] Mantovani M, Dos Santos Abel LD, Moreira-Filho O. Conserved 5S and variable 45S rDNA chromosomal localisation revealed by FISH in *Astyanax scabripinnis* (Pisces, Characidae). Genetica. 2005;123: 211–216. doi: 10.1007/s10709-004-2281-3 15954491 10.1007/s10709-004-2281-3

[74] Volker M, Sonnenberg R, Ráb P, Kullmann H. Karyotype differentiation in *Chromaphyosemion* killifishes (Cyprinodontiformes, Nothobranchiidae). III: extensive karyotypic variability associated with low mitochondrial haplotype differentiation in *C*. *bivittatum*. Cytogenet Genome Res. 2007;116: 116–26. doi: 10.1159/000097429 17268189 10.1159/000097429

[75] Gromicho M, Ozouf-Costaz C, Collares-Pereira MJ. Lack of correspondence between CMA_3_-, Ag-positive signals and 28S rDNA loci in two Iberian minnows (Teleostei, Cyprinidae) evidenced by sequential banding. Cytogenet Genome Res. 2005;109: 507–511. doi: 10.1159/000084211 15905646 10.1159/000084211

[76] Tymowska J, Fischberg M. A comparison of the karyotype, constitutive heterochromatin, and nucleolar organizer regions of the new tetraploid species *Xenopus epitropicalis* Fischberg and Picard with those of *Xenopus tropicalis* Gray (Anura, Pipidae). Cytogenet Cell Genet. 1982;34: 149–57. doi: 10.1159/000131803 7151486 10.1159/000131803

[77] Knytl M, Smolík O, Kubíčková S, Tlapáková T, Evans BJ, Krylov V. Chromosome divergence during evolution of the tetraploid clawed frogs, *Xenopus mellotropicalis* and *Xenopus epitropicalis* as revealed by Zoo-FISH. Cimini D, editor. PLoS One. 2017;12: e0177087 doi: 10.1371/journal.pone.0177087 28545147 10.1371/journal.pone.0177087 PMC5436656

[78] Jotterand M, Fischberg M. A chromosome mutation affecting the number of nucleoli in *Xenopus borealis* Parker. Experientia. 1974;30: 1003–1005. doi: 10.1007/BF01938973 10.1007/BF01938973 4411582

[79] Choleva L, Janko K. Rise and persistence of animal polyploidy: evolutionary constraints and potential. Cytogenet Genome Res. 2013;140: 151–70. doi: 10.1159/000353464 23838539 10.1159/000353464

[80] Lusk S, Hanel L, Lusková V. Red List of the ichthyofauna of the Czech Republic: development and present status. Folia Zool. 2004;53: 215–226.

[81] Economidis PS. Endangered freshwater fishes of Greece. Biol Conserv. 1995;72: 201–211. doi: 10.1016/0006-3207(94)00083-3

